# The READI European project: Enhancing inclusivity in clinical research

**DOI:** 10.1111/eci.70146

**Published:** 2025-11-17

**Authors:** Alberto M. Borobia, Juliette Guillot, Amanda Bok, Lea Proulx, Sandra Pla, Paloma Moraga, Ron Hillel, Silvia Bornengo, Bruno Jolain, Mónica García, Jose Luis Narro

**Affiliations:** ^1^ Clinical Pharmacology Department La Paz University Hospital, School of Medicine, Universidad Autónoma de Madrid, IdiPAZ Madrid Spain; ^2^ Novartis Basel Switzerland; ^3^ The Synergist Brussels Belgium; ^4^ F. Hoffmann‐La Roche Ltd. Basel Switzerland; ^5^ SYNAPSE Research Management Partners S.L Madrid Spain; ^6^ Clinical Pharmacology Department La Paz University Hospital, IdiPAZ Madrid Spain; ^7^ La Paz University Hospital Research Foundation. IdiPAZ Madrid Spain

**Keywords:** clinical studies, inclusivity, underserved and underrepresented populations

**Key Messages**

- Clinical studies must prioritize inclusive representativeness to ensure equitable healthcare outcomes and robust scientific evidence.
- Barriers to participation in clinical studies are multifaceted and require systemic and tailored solutions.
- The absence of standardized data descriptors and demographic criteria results in incomplete participant alignment and biases in recruitment, ultimately reducing the overall reliability of study findings.
- The READI project leverages digital innovation, new tools and designs, stakeholder engagement, and capacity building to transform the clinical research ecosystem in Europe to facilitate the participation of underserved and underrepresented populations, regardless of the disease and the environment.
- Long‐term sustainability and scalability are integral to the project's objectives, ensuring lasting impact beyond its duration.
- READI's collaboration with regulators and policymakers embeds inclusivity into the European clinical studies framework, setting a precedent for global adoption of inclusive clinical studies practices. Likewise, active stakeholder engagement, connection and education is key to achieving the project's objectives and driving systemic change.

## 1 INTRODUCTION

Clinical studies (CS) have often struggled to recruit and retain participants that represent the population who will ultimately receive the treatments. This lack of representation leads to gaps in understanding diseases, preventive factors and the safety and effectiveness of treatments across diverse populations.^1^ In this way, access to experimental health technologies—such as medicinal drugs, vaccines and medical devices—remains limited to specific populations.

Improving inclusiveness and representativeness in clinical studies is not just a matter of equity but a crucial step to ensure the quality and impact of clinical studies.^2^ Thus, broadening the scope of those who are included in clinical studies can be regarded as an attempt to ensure that innovations in healthcare are accessible to all, reducing disparities not only in Europe but across the globe. This approach will ultimately lead to more reliable data and more effective treatments that truly serve the needs of a diverse population.

Various factors are associated with health disparities, including but not limited to demographic characteristics such as ethnicity, sex, gender, socio‐economic status or age. Clinical studies must actively consider populations that have historically been underserved (US) and underrepresented (UR) in clinical studies. However, several barriers hinder participation, particularly among US and UR populations. These include geographic limitations, mistrust, restricted access to relevant information, ineffective communication, societal prejudices, financial constraints among other factors.^3^, ^4^

To fully understand their impact, it is essential to acknowledge the broader systemic challenges that complicate recruitment and retention of diverse participants in clinical studies. These challenges include:

- Participant recruitment and retention: Nearly 20% of randomized controlled trials are prematurely discontinued due to poor recruitment, often driven by factors such as mistrust, logistical constraints and limited cultural competences, among others, exacerbate these difficulties.^5^ Additionally, increasing protocol complexity and participant burden make long‐term commitment more difficult, especially for US and UR populations. Poor retention leads to missing data, weakening trial power and increasing sample size requirements. Recent studies indicate that up to 50% of all trials experience a loss to follow‐up of more than 11%.^6^ Ineffective retention strategies result in longer trials, higher costs, and expose additional participants to unnecessary risks.
- Barriers to access: Many clinical trial designs require participants to be physically present at specific study sites, restricting opportunities to participate for individuals in rural or underserved areas. Lack of awareness and limited dissemination of information about study availability and participation exacerbates this issue.
- Capacity gaps at clinical sites: Many clinical sites lack the expertise, resources and strategies necessary to effectively engage a broad range of participants. Additionally, both medical and non‐medical communities often lack the capacity to act as enablers or advocates in the recruitment process.
- Fragmentation of efforts: Existing training programs and tools designed to promote inclusivity are often disconnected and not tailored to specific audiences, failing to address the interrelated nature of these systemic barriers.

Addressing these challenges requires a fundamental transformation in the design and execution of clinical studies to ensure a truly representative patient population is identified and provided with equitable opportunities to participate. To drive this transformation, it is essential that relevant clinical study information is widely distributed and accessible to all populations, that professionals involved are properly trained and empowered to design, develop and manage innovative studies tailored to diverse populations, and that key stakeholders—including patients, caregivers and patient organizations—are actively involved in design, educated and engaged, as their participation is vital to the success of clinical studies.^7^

## 2 THE READI PROJECT: A HOLISTIC APPROACH TO INCLUSIVE CLINICAL STUDIES

In this challenging context, the Research in Europe and Diversity Inclusion (READI) Project aims to create a more integrated and democratic ecosystem for clinical studies by identifying barriers to inclusiveness and representativeness, setting a new standard for equity in clinical studies and fostering the empowerment of all stakeholders. Through this engaged ecosystem, stakeholders can provide and share innovative approaches, tools, training programs and valuable insights to facilitate reach, engagement, recruitment and retention of underserved and underrepresented participants, enhancing inclusiveness and representativeness in CS.

The READI Project will further accelerate and sustain its impact through an innovative digital platform designed to enhance access to clinical studies information and strengthen stakeholder connections. By adopting an intersectional and holistic approach, READI will ensure long‐term sustainability beyond the project's duration, ultimately transforming the way clinical studies are conducted in Europe.

Additionally, READI will align with various global clinical research initiatives, such as the WHO International Clinical Trials Registry Platform (ICTRP)^8^ and the Vulcan Accelerator,^9^ among others.

## 3 THE READI CONSORTIUM

**FIGURE 1:**
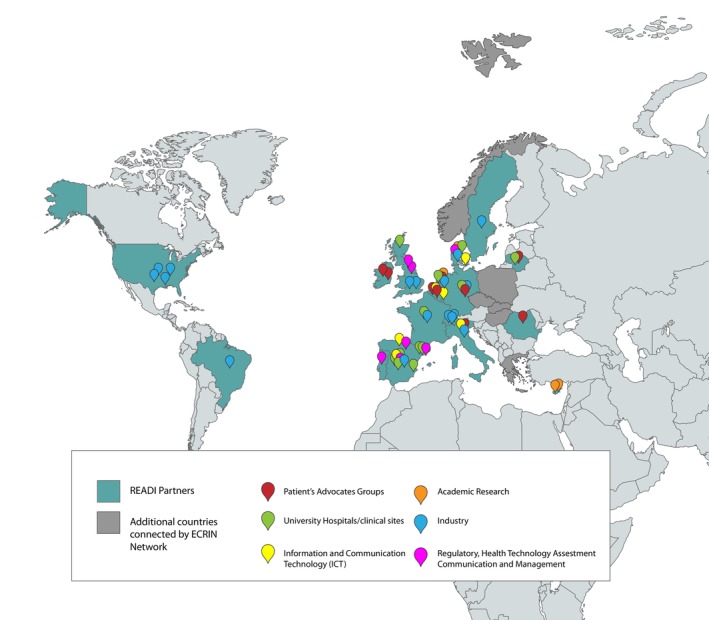
READI participants.

To achieve its ambitious objectives, READI has assembled one of the largest consortia ever formed for a European clinical studies initiative.^10^ Comprising 73 organizations from 18 countries: Belgium, Cyprus, Denmark, France, Germany, Ireland, Italy, Lithuania, Luxembourg, the Netherlands, Portugal, Romania, Spain, Sweden, Switzerland, the United Kingdom, the USA and Brazil (Figure 1). These organizations cover the full range of stakeholders involved in clinical research: patient organizations (with the largest participation ever in such a project), academic research centers, hospitals, technology companies, the European Federation of Pharmaceutical Industries and Associations (EFPIA), regulatory bodies, ethics experts, Health Technology Assessment and clinical research professionals.

**FIGURE 2:**
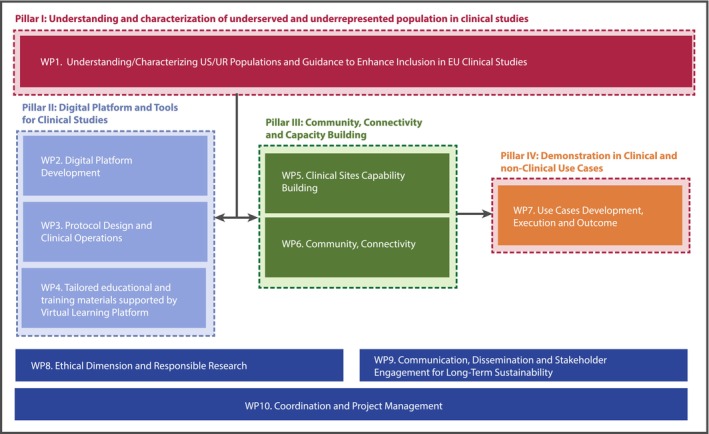
READI structure and organization.

With a budget of 66.8 million euros, this multidisciplinary consortium fosters cross‐collaboration to ensure that all aspects of clinical research—from study design to implementation—reflect the principles of inclusivity and equity, whereby everyone has access to innovation in healthcare. Additionally, the project is structured around 4 main pillars and 10 interconnected work packages (WPs), each addressing a critical component of the project's objectives (Figure 2):

1. Pillar I: Understanding and characterization of Underserved and Underrepresented in clinical studies is included in WP1. A comprehensive analysis will be conducted to identify key differentiators such as age, sex, gender, LGBTQI status, disabilities, refugee status, socio‐economic background and ethnicity among others. These factors will help characterize US and UR populations in European clinical studies. A thorough investigation of real‐world data (RWD) sources will characterize these populations in Europe and identify barriers to their inclusion in CS. Regulatory and Health Technology Assessment bodies (HTA) will be engaged to establish standardized evaluation methods for representative populations, ensuring transparency and inclusivity in CS policies. A working group of regulatory experts will develop recommendations to guide national agencies, contributing to initiatives such as ACT‐EU.^11^
2. Pillar II: Digital platform and tools for clinical studies. This pillar encompasses three work packages:
  - WP2: Digital Platform Development. READI will develop an open federated digital innovation platform to improve access to structured and enriched CS data. Key features include a unified data infrastructure, standardize data acquisition, AI‐driven data parsing, and community‐driven validation processes. The platform will facilitate data accessibility, improve patient recruitment and support diversity‐focused study designs. Efforts will ensure cultural and linguistic inclusivity, implementing accessibility standards for individuals with disabilities.
  - WP3: Protocol Design and Clinical Operations will focus on identifying, assessing and further developing innovative methods and tools for inclusive clinical study design, including diversity components in protocol analysis, recruitment strategies and pragmatic and decentralized approaches.
  - WP4: Tailored educational and training materials supported by Virtual Learning Platform will identify knowledge and existing training gaps related to the inclusion of underserved and underrepresented populations in CS and develop tailored educational programs for key stakeholders such as clinical researchers, healthcare providers and patient organizations. A Virtual Learning Platform (VLP) will be established to consolidate training resources.
3. Pillar III: Community Connectivity and Capacity Building. This pillar includes two work packages:
  - WP5: Clinical Studies Capability Building. READI will strengthen the competencies of clinical sites to better engage with underrepresented populations and increase their participation in clinical studies. This involves identifying new clinical sites and expanding existing European Clinical Trial Site Networks (as VACCELERATE Site Network^12^ or Connect4Children^13^), assessing knowledge gaps, and implementing tailored training programs as needed. Additionally, READI will establish international structures to support underrepresented countries in multicenter clinical studies, addressing geographical inconsistencies that may act as proxies for US and UR populations.
  - WP6: Community Connectivity. READI aims to strengthen stakeholder relationships and promote community engagement clusters to address barriers and mistrust in clinical studies, particularly focusing on patients, caregivers and healthcare professionals. This includes targeted outreach to diverse populations based on geography, culture, comorbidities, gender, age, sex and other relevant characteristics.
4. Pillar IV: Demonstration in Clinical and Non‐Clinical Use Cases, is led by WP7. The READI digital platform, tools and methodologies developed will be assessed in at least four clinical use cases proposed by the industry partners acting as sponsors and in collaboration with READI partners. The main activities in this pillar include identifying the most suitable clinical studies and evaluating their suitability—considering the involvement of underserved and underrepresented populations in targeted disease areas—as well as coordinating the implementation of the use cases, providing support for the integration of READI solutions and analysing their performance and outcomes to validate the approach.

In addition to these pillars, READI includes three transversal work packages (WP8, WP9 and WP10) aimed at ensuring ethical compliance, data privacy, regulatory alignment, sustainability and project management:

- WP8: Ethical Dimension and Responsible Research—Guarantees adherence to the highest ethical and regulatory standards, ensuring compliance with GDPR (General Data Protection Regulation) and Good Clinical Practices.
- WP9: Communication, Dissemination and Stakeholder Engagement for Long‐Term Sustainability—Ensures the long‐term viability of project outcomes by defining strategies for knowledge dissemination, stakeholder engagement and regulatory alignment. It also develops comprehensive communication strategies to enhance the visibility and impact of READI initiatives among key stakeholders and the general public.
- WP10: Coordination and Project Management—Provides scientific and technical guidance, and professional project management to ensure adequate progress and successful completion of the project.

All these work packages progress in parallel and are led by a combination of academic institutions, patient representatives, civil society and EFPIA members, ensuring a balanced collaboration between the public and private sectors (Table 1), all of them participating in the Steering Committee. The overall coordination of the project is managed by Alberto Borobia (SERMAS) as Coordinator and Juliette Guillot (Novartis) as Project Leader, with the support of Amanda Bok (The Synergist) as Digital and Sustainability Coordinator. This structure ensures the proper development, WP interconnection and collaborative work among all project partners, guaranteeing the success and achievement of its objectives.

**TABLE 1:** WP leaders and co‐leaders.

WP leaders	Non‐industrial beneficiaries	Industrial beneficiaries and contributing partners
WP1	EUC: Theodore Lytras UNIC: Zoi‐Dorothea Pana	MHRA: Rachael Williams Pfizer: Solomon Makgoeng J&J
WP2	ITTM: Andreas Kremer, Gabriel Sieglerschmidt Synergist: Nersey Rastan	AZ: John Rivers J&J
WP3	ECRIN: Amelie Michon	Roche: Bruno Jolain
WP4	EUPATI: Maria Dutarte	GSK: Michel Reid
WP5	UHC: Oliver Andreas Cornely, Kerstin Albus	AbbVie: Sasha Tyndale
WP6	Synergist: Derick Mitchell	Roche: Lea Proulx AZ: Barbara Valastro
WP7	SERMAS: Irene Garcia	Novartis: Juliette Guillot Pfizer: Leo Russo
WP8	SERMAS (FIBHULP): Cristina Murano	Sanofi: Monique Adams, Susan Tio
WP9	Synergist: Derick Mitchell	BREAKTHROUGHT1D: Carmen Hurtado del Pozo TPIZ: Akiko Otani, Adam MacNeill
WP10	SERMAS: Alberto Borobia	Novartis: Ron Hillel

## 4 IMPACT OF THE PROJECT

As reflected in the previous lines, the READI project will transform the way clinical studies are conducted in Europe. It is designed to transform clinical trial ecosystems and capabilities, with patients at the center, and key stakeholders collaborating from the design phase to the delivery phase. In this way, the knowledge generated will not only be beneficial by itself, but also useful for making recommendations to advance inclusive representation in CS and develop ethical standards. It will provide an innovative methodology to recruit underserved and underrepresented populations, as well as a change in CS communication by expanding a network of sites capable of conducting CS across Europe, by equipping diverse entities with the necessary capabilities to recruit and retain US and UR populations.

Specifically, it is expected to expand the understanding of underserved and underrepresented populations, leading to a 60% increase in CS recruitment representativeness compared to historical data. In doing so, an agreed set of data standards for demographic descriptors will be developed, along with a toolbox for patient recruitment that includes digital platforms reaching at least 100 organizations. Testing these tools is crucial, so at least four clinical use cases will be developed to demonstrate their impact and effectiveness.

## 5 DISCUSSION

The READI project addresses critical systemic barriers to inclusivity in clinical studies, such as geographical limitations or mistrust. By leveraging a holistic, intersectional approach, READI aims to democratize access to clinical studies and enhance the representation of US and UR populations in CS. Key innovations, such as the AI‐powered digital platform and the development of a standardized set of descriptors for US and UR populations, will enhance recruitment efficiency, improve retention rates, and increase the generalizability of clinical study findings. To further support patient engagement, community clusters and targeted educational activities will help address participation barriers. Additionally, the focus on capacity building ensures that clinical sites across Europe are equipped with the necessary skills and tools to reach and engage with US and UR populations effectively. The implementation of use cases provides a practical framework for validating these innovations and generating actionable insights that will support long‐term sustainability.

The READI project represents a paradigm shift in the conduct of clinical research in Europe, enhancing inclusivity and equity. By addressing the systemic barriers that limit the participation of underserved and underrepresented populations, READI has the potential to transform clinical studies into being more representative and impactful. The outcomes of this project will not only help establish ethical, effective and inclusive CS practices across Europe and beyond, but also enhance access to innovations in healthcare. Future research should build on these achievements to refine methodologies and promote global adoption of inclusive CS standards.

## AUTHOR CONTRIBUTIONS

Alberto M. Borobia: Project coordination, conceptualization, supervision of manuscript preparation and critical review. Juliette Guillot: Project leadership, consortium coordination and drafting of key sections. Amanda Bok, Léa Proulx, Ron Hillel, Bruno Jolain, Sandra Pla, Mónica García, Paloma Moraga, Jose Luis Narro, and Silvia Bornengo: Provided content expertise, contributed to writing and reviewing specific sections related to their institutional work packages. All authors contributed to the manuscript, reviewed the final version and approved it for submission.

## FUNDING INFORMATION

The project leading to this publication is supported by the Innovative Health Initiative Joint Undertaking (IHI JU) under grant agreement No. 101166227. The JU receives support from the European Union's Horizon Europe research and innovation programme and COCIR, EFPIA, EuropaBio, MedTech Europe, Vaccines Europe, and Medicines and Healthcare products Regulatory Agency and Breakthrough T1D. This project is also supported by UKRI (UK Research and Innovation) grant agreement No 10152425 for National Institute for Health and Care Excellence and grant agreement No 31052024 for The University Court of the University of Aberdeen. Webpage: https://ihi‐readi.org.

## CONFLICT OF INTEREST STATEMENT

The authors declared no competing interests for this work.

## DISCLAIMER

This publication is organized and funded by the IHI READI consortium members. This publication is intended for non promotional scientific purposes only and may contain information on products or indications currently under investigation and/or that have not been approved by the regulatory authorities. This publication is accurate at the time of presentation. The content of this publication is not intended to establish any legally enforceable rights, obligations or commitments on the IHI READI consortium members.

## Data Availability

The data presented in this article are based on project documentation and consortium contributions from the READI European initiative. Data supporting the findings are available from the corresponding author upon reasonable request. Due to privacy and consortium agreements, some datasets are not publicly available.
